# Fibrinogen performs better than D-dimer for the diagnosis of periprosthetic joint infection: a meta-analysis of diagnostic trials

**DOI:** 10.1186/s13018-020-02109-3

**Published:** 2021-01-09

**Authors:** Liping Pan, Hao Wu, Heng Liu, Xin Yang, Zhichao Meng, Yongping Cao

**Affiliations:** Department of Orthopedics, Peking University First Hospital, No. 8 Xishiku Street, XiCheng District, Beijing, 100034 People’s Republic of China

**Keywords:** Periprosthetic joint infection, D-dimer, Fibrinogen, Diagnosis, Meta-analysis

## Abstract

**Purpose:**

D-dimer and fibrinogen, both belonging to coagulation parameters, are controversial for the diagnosis of periprosthetic joint infection (PJI). This meta-analysis was conducted to compare their diagnostic accuracies for PJI by synthesizing currently available evidence.

**Methods:**

Cochrane Library, MEDLINE, Web of Science, and Embase up to March 1, 2020, and other relevant articles were searched. Five hundred and eighty-one articles were identified after initial research, and 11 studies were included finally. No threshold effects were found between studies. The pooled sensitivity, specificity, and positive and negative likelihood ratio were reported to evaluate the diagnostic performance with heterogeneity analysis. *Z* test statistics was used to analyze the difference of diagnostic performance between D-dimer and fibrinogen.

**Results:**

The pooled sensitivity, specificity, and positive and negative likelihood ratio of D-dimer for PJI were 0.79 (95% [CI], 0.72–0.85), 0.77 (0.67–0.84), 3.38 (2.21–5.18), and 0.27 (0.18–0.41), respectively. As for fibrinogen, the pooled sensitivity, specificity, and positive and negative likelihood ratio for PJI were 0.75 (0.68–0.80), 0.85 (0.82–0.88), 5.12 (4.22–6.22), and 0.30 (0.23–0.37), respectively. Great heterogeneity was found in studies for D-dimer, and univariate meta-regression analysis revealed that number of involved joints, disease spectrum, comorbidities influencing D-dimer, and sample sources were the source of heterogeneity. *Z* test found that the pooled specificity of fibrinogen was significantly higher than D-dimer (0.85 ± 0.01 versus 0.77 ± 0.04, *p* = 0.03). The pooled positive likelihood ratio of fibrinogen was significantly higher than D-dimer (5.12 ± 0.51 versus 3.38 ± 0.74, *p* = 0.03).

**Conclusion:**

Based on currently available evidence, the meta-analysis suggests that fibrinogen performs better than D-dimer as a rule-in diagnostic tool for its higher specificity. However, more prospective trials with larger size are still needed to provide further confirmation.

**Trial registration:**

This meta-analysis was prospectively registered on PROSPERO (International prospective register of systematic reviews), and the registering number was CRD42020177176.

## Introduction

Periprosthetic joint infection (PJI) is a rare but devastating complication for patients undergoing joint arthroplasty [1]. It has a huge impact on the joint function and quality of life of patients, causes significant morbidity, and accounts for a substantial proportion of health care expenditure [2]. The Musculoskeletal Infection Society (MSIS) diagnostic criteria [3] for PJI of hips and knees, slightly revised in 2013 by the International Consensus Meeting (ICM) [4], has obtained widespread recognition.

Saxena et al. [5] first discovered an association between coagulopathy and PJI, and some studies extended the research targeting D-dimer and fibrinogen as potential tools for PJI diagnosis. Circulating D-dimer is the characteristic product of degradation of cross-linked fibrin, and fibrinogen is a protein for blood coagulation produced primarily by hepatocytes, both of which could be assessed easily and conveniently in coagulation measurements. Based on the previous research, the new evidence-based 2018 criteria [6] for PJI were published with a better diagnostic accuracy, in which a minor criterion of serum D-dimer was added.

However, some studies [7–17] have demonstrated controversial diagnostic accuracy of D-dimer and fibrinogen as a single biomarker for PJI recently. Some showed that D-dimer was not accurate enough to distinguish PJI and aseptic loosening [8], and others believed fibrinogen could perform better than plasma D-dimer for PJI diagnosis [15], though D-dimer was minor criteria and fibrinogen was not in the new evidence-based 2018 criteria [6] for PJI.

Thus, this meta-analysis aimed to explore the accuracies of D-dimer and fibrinogen for the diagnosis of PJI via synthesizing current available evidence. We sought to determine whether fibrinogen performed better than D-dimer for PJI diagnosis.

## Methods and materials

### Search strategy and criteria

A systematic approach as required following the Preferred Reporting Items for Systematic Reviews and Meta-Analyses (PRISMA) guidelines [18] was used. A protocol was registered previously online with PROSPERO (International prospective register of systematic reviews), as recommended by PRISMA, and the registration number was CRD42020177176. Cochrane Library, MEDLINE, Web of Science, and Embase from inception up to March 1, 2020, were searched. Keywords or mesh words used were as follows: (“prosthesis-related infections” OR “periprosthetic joint infection” OR “prosthetic infection” OR “joint prosthesis” OR “arthroplasty” OR “replacement”) AND “infection” AND (“D-dimer” OR “fibrinogen”). Other relevant articles and their bibliographies were searched in a manually manner after the initial search. It was restricted to publications in English.

### Inclusion and exclusion criteria

**Fig. 1:**
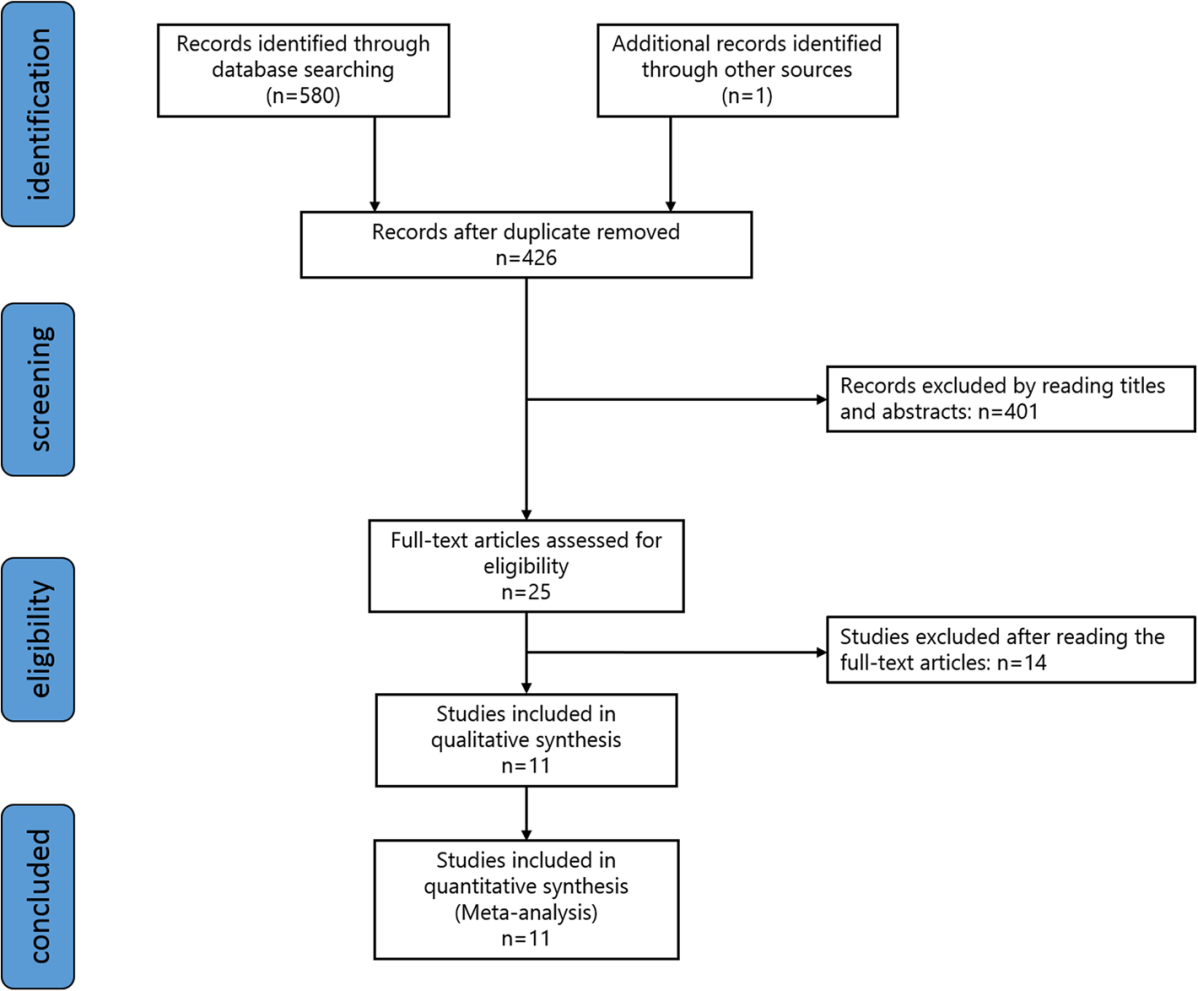
Flow diagram showing the study screening process

The inclusion criteria for studies included patients who underwent joint arthroplasties, confirmed PJI by the MSIS or AAOS guidelines, D-dimer or fibrinogen detected before revision, and sufficient data for sensitivity and specificity. Exclusion criteria included case reports, expert opinions, animal experiments, reviews, unrelated biomarkers, duplications, publications in other languages, and not sufficient data to extract. Two independent reviewers (LP and HW) who were board-certified orthopedic surgeons conducted the screening of all of the studies. Any discrepancy was reported to a third reviewer for a final decision. A total of 581 records were identified after initial screening. One hundred and fifty-five duplicates were removed, and 415 were excluded after filtering through titles, abstracts, and full texts. Eventually, a total of 11 eligible studies were included in qualitative and quantitative analysis [7–17]. A PRISMA flow diagram of screening studies through this meta-analysis is provided in Fig. 1.

### Assessment of study quality

Totally, 1465 subjects were included in studies for D-dimer, and 1039 subjects were included in studies for fibrinogen. Of these 11 studies, 4 were prospective studies, while the remaining 7 were retrospective. The average age of the subjects ranged from 61.60 to 68.89 years old. Among these 11 studies, 9 studies used D-dimer for PJI diagnosis, 4 used fibrinogen, and 2 evaluated both D-dimer and fibrinogen in the same trial. Except two subjects in one study had other joints involved [7], all other subjects in the studies had only knee and hip arthroplasties involved. The detailed clinical and methodological characteristics are shown in Table 1.

**Table 1:** Characteristics of included studies

Study	Country	Study design	Gender (F/M)	Age (years ± SD)	Involved joints (PJI/total)	Sites of arthroplasty	Disease spectrum	Comorbidities *	Cutoff
D-dimer									
Qin et al. [11]	China	P	69/53	65.21 ± 10.51	55/122	Hips and knees	Chronic PJI + aseptic	Excluded	1170 ng/ml
Xiong et al. [13]	China	P	48/32	61.60 ± 12.22	26/80	Hips and knees	PJI + aseptic	Excluded	756 ng/ml
Shahi et al. [12]	USA	P	94/101	62.46	57/195	Hips and knees	Primary arthroplasty + PJI + aseptic + reimplantation	Not excluded	850 ng/mL
Xu et al. [14]	China	R	NM	NM	82/224	Hips and knees	PJI + aseptic	Excluded	1020 ng/mL
Huang et al. [8]	China	R	46/34	67.15 ± 2.64	31/101	Hips and knees	Primary arthroplasty + PJI + aseptic	Excluded	850 ng/mL
Pannu et al. [10]	USA	R	62/49	68.89 ± 10	49/111	Hips and knees	PJI + aseptic	NM	2300 ng/mL
Hu et al. [7]	China	R	41/36	63.08	40/77	Hips and knees, 2 others	PJI + aseptic	Not excluded	955 ng/ml
Li et al. [15]	China	R	NM	NM	76/439	Hips and knees	PJI + aseptic	Excluded	1250 ng/ml
Wu et al. [17]	China	R	82/54	66.32 ± 11.73	35/116	Hips and knees	PJI + aseptic+reimplantation	Not excluded	410 ng/ml
Fibrinogen									
Li et al. [15]	China	R	NM	NM	76/439	Hips and knees	PJI + aseptic	Excluded	4.01 g/L
Wu et al. [17]	China	R	82/54	66.32 ± 11.73	35/116	Hips and knees	PJI + aseptic+reimplantation	Not excluded	3.61 g/L
Klim et al. [16]	Austria	P	NM	65.50 ± 15.31	78/124	Hips and knees	PJI + aseptic	Excluded	5.19 g/L
Xu et al. [9]	China	R	NM	NM	153/360	Hips and knees	PJI + aseptic	Excluded	3.57 g/L

*NM* not mentioned, *PJI* periprosthetic joint infection, *P* perspective, *R* retrospective

*Comorbidities referred to comorbidities influencing D-dimer or fibrinogen

**Fig. 2:**
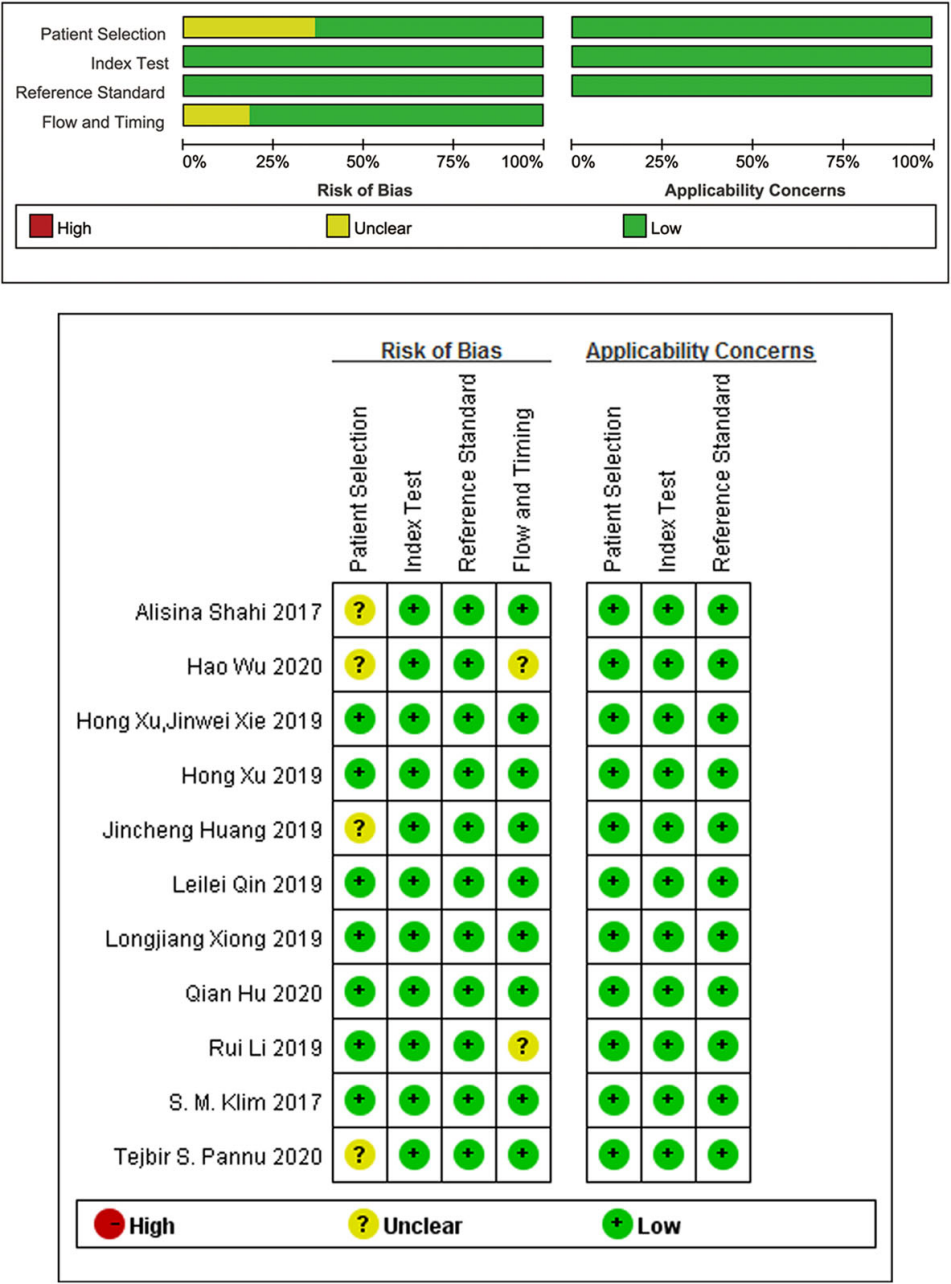
Grouped bar charts showing the risk of bias and applicability concerns for the 11 included studies according to the QUADAS-2 tool

The quality assessment was evaluated using the QUADAS-2 tool [19] as shown in Fig. 2. Two independent reviewers (LP and HW) conducted the screening of all of the studies. Any discrepancy was reported to a third reviewer for a final decision. Four studies [8, 10, 12, 17] were considered to have unclear risk of bias in patient selection due to improper patient inclusion criteria in original trials. Two studies [15, 17] were considered to have unclear risk of bias in the flow and timing due to not including all consecutive patients.

### Data collection and extraction

Two independent reviewers (LP and HW) extracted the data using standardized spreadsheets and were blinded to the studies’ institutions during the extraction of data process. Any discrepancy was reported to a third reviewer for a final decision. The data extracted included first author, publication year, country, study design, gender, size of involved joints, sites of arthroplasty, exclusion criteria, disease spectrum, optimal cutoff value of the tests, sensitivity, specificity, or true positive (tp), false positive (fp), false negative (fn), true negative (tn) values if provided, and the referenced gold-standard tests.

### Statistical analyses

Spearman’s correlation coefficient was used to assess a possible threshold effect because different studies might have used different cutoff values, which greatly influenced the estimation of sensitivity and specificity. The threshold for D-dimer and fibrinogen was mentioned in all studies, as shown in Table 1. No threshold effect (suggested by a strong positive correlation) was found in studies for D-dimer (Spearman’s correlation coefficient − 0.048; *p* = 0.911) and fibrinogen (Spearman’s correlation coefficient 0.40; *p* = 0.60).

Without threshold effect, statistics for diagnostic accuracy of D-dimer and fibrinogen for PJI including sensitivity, specificity, positive likelihood ratio (LR+), and negative likelihood ratio (LR−) were calculated. Heterogeneity was estimated using the *I*^2^ statistic. *I*^2^ values between 0 and 50% indicated minor heterogeneity. Values of > 50% indicated substantial heterogeneity. Meta-regression and subgroup analyses were conducted to identify potential sources of bias, such as country, study design, number of involved joints, disease spectrum, comorbidities influencing D-dimer, sample source, and optimal cutoff value by receiver operating characteristic (ROC) curve, where substantial heterogeneity was found. The software Stata version 15 and the midas commands were utilized for all analyses. Publication bias was assessed using the methods provided by Deeks et al. [20]. The Deeks funnel plots and regression tests indicated a low possibility of publication bias in studies for D-dimer and fibrinogen (*p* = 0.21 and *p* = 0.68 for D-dimer and fibrinogen, respectively). The difference of the pooled sensitivity, specificity, LR+, and LR− between D-dimer and fibrinogen was analyzed using *Z* test statistics, and *p* value of < 0.05 was considered significant.

## Results

### Diagnostic performance of D-dimer for PJI

**Fig. 3:**
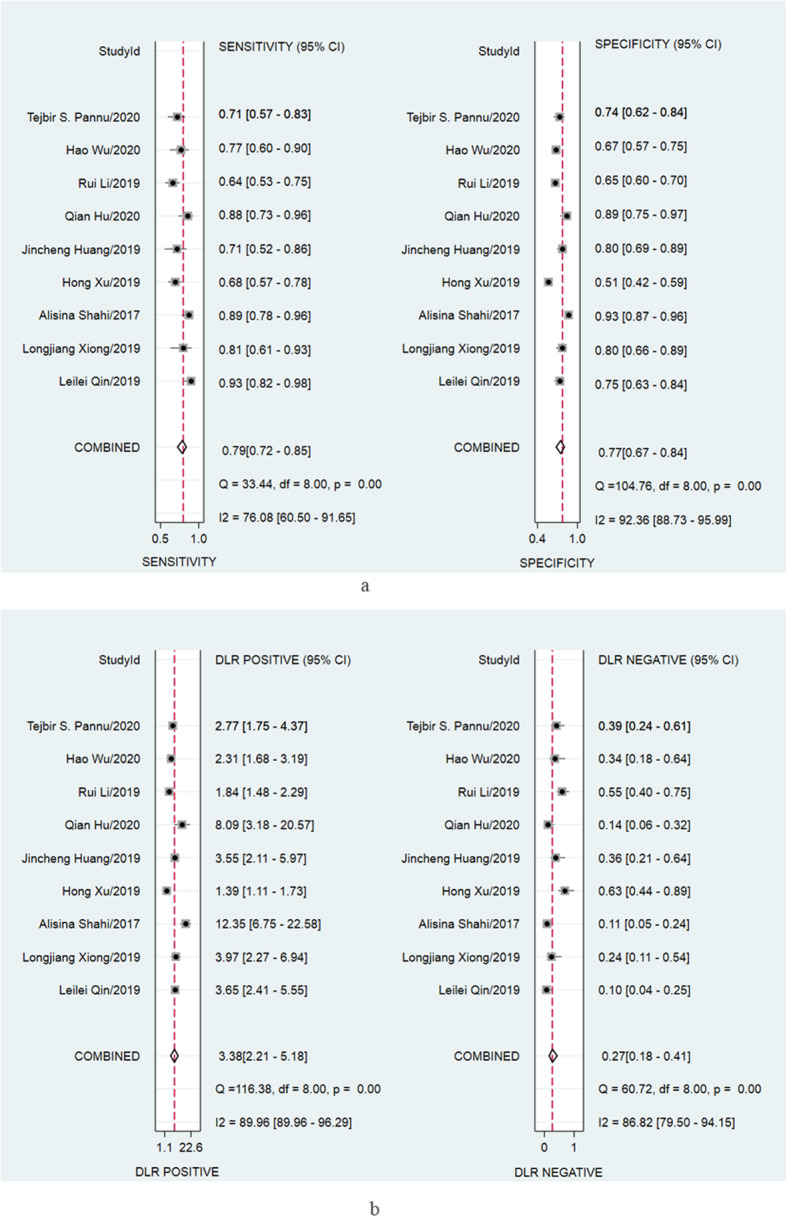
Paired forest plots of the sensitivity and specificity (**a**) and positive and negative likelihood ratio (**b**) of D-dimer for the diagnosis of PJI

Nine studies [7, 8, 10–15, 17] including 1465 subjects were synthesized for the diagnosis performance of D-dimer for PJI. The pooled sensitivity and specificity of D-dimer for PJI were 0.79 (95% confidence interval [CI], 0.72 to 0.85) and 0.77 (95% CI, 0.67 to 0.84), respectively (Fig. 3a). Substantial heterogeneity was identified among studies as the *I*^2^ values for sensitivity and specificity were 76.08% (95% CI, 60.5 to 91.65%) and 92.36% (95% CI, 88.73 to 95.99%), respectively. The pooled positive and negative likelihood ratio of D-dimer for PJI were 3.38 (95% [CI], 2.21 to 5.18) and 0.27 (95%CI, 0.18 to 0.41), respectively (Fig. 3b). The *I*^2^ values for positive and negative likelihood ratio were 89.96% (95% CI, 89.96 to 96.29%) and 86.82% (95% CI, 79.50 to 94.15%), respectively.

### Diagnostic performance of fibrinogen for PJI

**Fig. 4:**
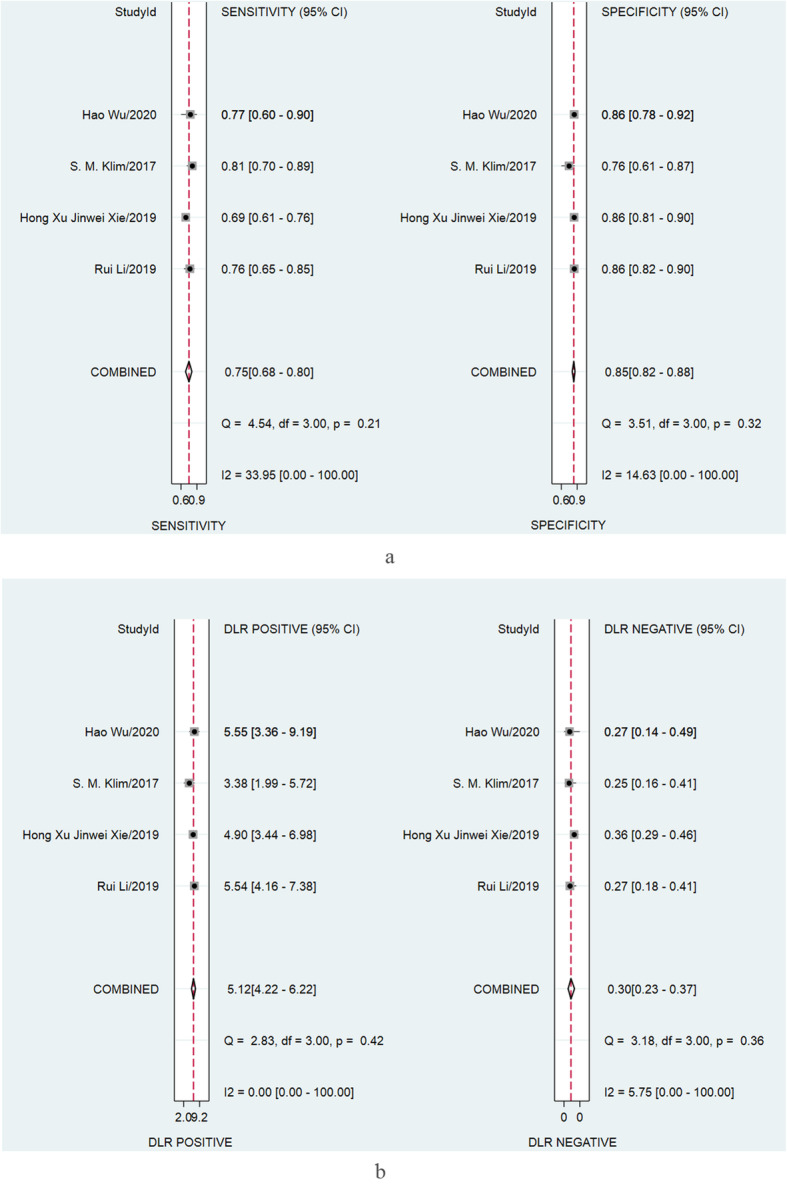
Paired forest plots of the sensitivity and specificity (**a**) and positive and negative likelihood ratio (**b**) of fibrinogen for the diagnosis of PJI

Four studies [9, 15–17] including 1039 subjects were synthesized for the diagnosis performance of fibrinogen for PJI. The pooled sensitivity and specificity of fibrinogen for PJI were 0.75 (95% [CI], 0.68 to 0.80) and 0.85(95%CI, 0.82 to 0.88), respectively (Fig. 4a). Minor heterogeneity was identified among studies as the *I*^2^ values for sensitivity and specificity were 33.95% (95% CI, 0 to 100%) and 14.63% (95% CI, 0 to 100%), respectively. The pooled positive and negative likelihood ratio of fibrinogen for PJI were 5.12 (95% [CI], 4.22 to 6.22) and 0.30 (95% CI, 0.23 to 0.37), respectively (Fig. 4b). The *I*^2^ values for positive and negative likelihood ratio were 0% (95% CI, 0 to 100%) and 5.75% (95% CI, 0% to 100%), respectively.

### Meta-regression and subgroup analysis

Heterogeneity was identified for pooled sensitivity and specificity among studies of D-dimer and fibrinogen. Substantial heterogeneity was found in studies for D-dimer, and minor heterogeneity for fibrinogen. Therefore, univariate meta-regression and subgroup analysis were performed to identify potential sources of heterogeneity among studies for D-dimer. The results of the meta-regression and subgroup analysis are shown in Table 2. The significant sources of heterogeneity in terms of sensitivity were number of involved joints, comorbidities influencing D-dimer, and sample source. The significant sources of heterogeneity in terms of specificity were disease spectrum, number of involved joints, and comorbidities influencing D-dimer. Other factors, like country, study design, and optimal cutoff values were not significantly different.

**Table 2:** Univariable meta-regression and subgroup analysis for potential heterogeneity in studies for D-dimer

Covariate	No. of studies	Sensitivity (95% CI)	***P*** value	Specificity (95% CI)	***P*** value
**Country**					
USA	2	0.82 [0.69–0.95]	0.27	0.86 [0.75–0.97]	0.90
China	7	0.79 [0.71–0.87]	.	0.73 [0.64–0.82]	.
**Study design**					
Prospective	3	0.89 [0.83–0.95]	0.41	0.84 [0.75–0.94]	0.71
Retrospective	6	0.73 [0.67–0.79]	.	0.72 [0.61–0.82]	.
**No. of involved joints**					
> 150	3	0.76 [0.64–0.87]	0.01*	0.74 [0.58–0.89]	0.14
< 150	6	0.81 [0.74–0.89]	.	0.78 [0.68–0.88]	.
**Disease spectrum**					
PJI + aseptic	6	0.79 [0.70–0.87]	0.06	0.73 [0.62–0.84]	0.03*
PJI + aseptic + primary arthroplasty/reimplantaion	3	0.81 [0.69–0.92]	.	0.82 [0.71–0.93]	.
**Comorbidities influencing D-dimer**					
Excluded	5	0.77 [0.67–0.86]	0.01*	0.71 [0.59–0.82]	0.01*
Not excluded	4	0.82 [0.73–0.91]	.	0.83 [0.73–0.92]	.
**Sample source**					
Plasma	4	0.75 [0.65–0.85]	0.00*	0.68 [0.56–0.81]	0.00*
Serum	5	0.83 [0.75–0.90]	.	0.82 [0.74–0.90]	.
**Optimal cutoff value**					
< 900 ng/ml	4	0.81 [0.71–0.91]	0.14	0.81 [0.72–0.91]	0.54
> 900 ng/ml	5	0.78 [0.69–0.87]	.	0.72 [0.60–0.83]	.

*Statistical significance (*p* < 0.05)

### The difference of pooled diagnostic performance between D-dimer and fibrinogen

*Z* test statistics was conducted to analyze the difference of the pooled diagnostic performance between D-dimer and fibrinogen, as shown in Table 3. The pooled sensitivity of D-dimer and fibrinogen was 0.79 ± 0.04 (value ± standard error) and 0.75 ± 0.03, and no significant difference was found as *p* value was 0.15. The pooled specificity of D-dimer and fibrinogen was 0.77 ± 0.04 and 0.85 ± 0.01, and significant difference was found for *p* value less than 0.05. The pooled positive likelihood ratio of D-dimer and fibrinogen was 3.38 ± 0.74 and 5.12 ± 0.51, and significant difference was found for *p* value less than 0.05. The pooled negative likelihood ratio of D-dimer and fibrinogen was 0.27 ± 0.06 and 0.30 ± 0.04, and no significant difference was found as *p* value was 0.34.

**Table 3:** Comparison of pooled diagnostic performance of D-dimer and fibrinogen by Z-test statistics

	D-dimer			Fibrinogen			Z value	*p* value
	Pooled value	SE	95% CI	Pooled value	SE	95% CI		
Sensitivity	0.79	0.04	0.72–0.85	0.75	0.03	0.68–0.80	1.02	0.15
Specificity	0.77	0.04	0.67–0.84	0.85	0.01	0.82–0.88	1.93	0.03^*^
LR+	3.38	0.74	2.21–5.18	5.12	0.51	4.22–6.22	1.94	0.03^*^
LR−	0.27	0.06	0.18–0.41	0.30	0.04	0.23–0.37	0.42	0.34

*SE* standard error, *CI* confidence interval, *LR+* positive likelihood ratio, *LR−* negative likelihood ratio

*p value less than 0.05 meant significant difference

## Discussion

Timely and accurate diagnosis of PJI of the hips and knees remains a major challenge for orthopedic surgeons as there are no absolutely accurate tests currently [21, 22]. Numerous biomarkers have been discovered and become available in the recent years, including circulating D-dimer and fibrinogen in coagulation system. D-dimer and fibrinogen could be detected in coagulation test which was essential preoperative procedures. Thus, no additional costs would be incurred if D-dimer and fibrinogen were taken as regular diagnostic tools. This may be an incentive for clinicians to use them more routinely. This meta-analysis aimed to compare the diagnostic accuracy of D-dimer and fibrinogen from the available published studies. Our pooled results showed that D-dimer and fibrinogen performed well in sensitivity and specificity in diagnosing PJI, and fibrinogen achieved a significantly higher specificity. Fibrinogen had a higher positive likelihood ratio to be qualified as a rule-in diagnostic tool. Both biomarkers had a low negative likelihood ratio, making them suitable to be qualified as rule-out tools. Overall, as a single test, both of them could be used for PJI diagnosis, while fibrinogen had a higher diagnostic value.

This study had a number of limitations. First, not all of the sample sizes were big enough, and most of the studies came from the same region, and were not prospective. These may affect the quality of the trials and might have caused additional bias. Second, although low possibility of publication bias (*p* > 0.05) was observed by Deeks’ funnel plots, there was still some extent of publication bias because positive results tend to be more likely to be published. Third, though D-dimer and fibrinogen could be used for PJI diagnosis, the optimal cutoff values were not identified, which was of great importance for clinical applications and could not be resolved by meta-analysis. Fourth, though D-dimer and fibrinogen were considered as good individual biomarkers for PJI, combined tests of D-dimer or fibrinogen with other biomarkers like ESR or CRP might perform even better, which was to be confirmed through further prospective trials.

Both D-dimer and fibrinogen showed high sensitivity and specificity, which indicated that both could be used for clinic practice for PJI diagnosis. A guideline defines that LR+ > 2, LR− < 0.5 is considered a viable predictor, and LR + > 5, LR − < 0.2 is considered a good predictor [23]. Great heterogeneity was found in studies for D-dimer, and univariate meta-regression analysis revealed that number of involved joints, disease spectrum, comorbidities influencing D-dimer, and sample sources were the source of heterogeneity. As for studies for fibrinogen, minor heterogeneity was found.

D-dimer is a characteristic degradation product of cross-linked fibrin [24]. It is an established screening test for thrombotic diseases and extended its unconventional use to inflammation and infection, especially to PJI. It was proved that D-dimer had a significant differentiating ability in sepsis-related mortality [25]. D-dimer had been considered as one of the minor criteria for its diagnostic accuracy in the newly established evidence-based PJI definition [6]. Although Xu et al. [14] and Huang et al. [8] demonstrated that D-dimer had limited value and was not suitable for PJI diagnosis, our pooled results embodied these two studies showed that D-dimer had a high sensitivity and specificity to be a diagnostic tool for PJI, which were consistent with the established criteria.

Fibrinogen, a protein for blood coagulation, is synthesized by hepatocytes [26]. While its key role in the coagulation cascade is general knowledge, fibrinogen also plays a pivotal role in regulating inflammation process and preventing infection [27]. Our results showed that fibrinogen had a high sensitivity and specificity for PJI, which was consistent with the results exploring fibrinogen and D-dimer for PJI in the same trial [15, 17]. Thus, it could also be used as a biomarker for PJI. Many studies exploring the connection between fibrinogen and infection found reduced fibrinogen lead to compromised pathogen clearance, exacerbated pathogen infection, and increased mortality following subcutaneous infection [28, 29]. The underlying mechanism for connection between fibrinogen and PJI is still to be explored.

Substantial heterogeneity was found in studies for D-dimer, and its potential sources were explored by performing meta-regression and subgroup analysis. The results showed that number of involved joints, disease spectrum, comorbidities influencing D-dimer, and sample source were sources of heterogeneity. Comorbidities like inflammatory arthritis, deep vein thrombosis, a prosthetic heart valve, a history of hypercoagulation disorder, or other infectious diseases would influence the level of D-dimer [11]. The results found higher sensitivity and specificity would be achieved when all these comorbidities were excluded. In order to get a more accurate diagnosis, these comorbidities should be carefully assessed. The meta-regression results revealed that serum D-dimer performed better than plasma levels in the diagnosis of PJI. Serum D-dimer was measured after blood clotting with consumption of fibrinogen and some other coagulation factors, which would be different from plasma D-dimer levels. Previous studies did not have a uniform conclusion about the relationship between plasma and serum D-dimer [30–32]. More studies could be conducted to further exploring the difference.

*Z* test statistics found the pooled specificity and LR+ of fibrinogen were significantly higher than D-dimer, although no significant difference of sensitivity and LR− was found between fibrinogen and D-dimer. These indicated that fibrinogen performed better than D-dimer for PJI diagnosis. These results were consistent with other studies exploring the difference of D-dimer and fibrinogen in PJI diagnosis [15, 17]. As we know, D-dimer is included as one minor criterion in the newly established diagnosis criteria of PJI in 2018 [6]. Fibrinogen could be another better criterion for PJI diagnosis, and more prospective and larger-sized trials should be conducted to confirm it.

## Conclusion

Based on currently available evidence, the meta-analysis suggests that fibrinogen performs better than D-dimer as a rule-in diagnostic tool for its higher specificity. However, more prospective trials with larger size are still needed to provide further confirmation.

## Data Availability

All data and materials are contained within the manuscript.

## Abbreviations

PJI: Periprosthetic joint infection
MSIS: The Musculoskeletal Infection Society
ICM: International Consensus Meeting
PRISMA: The Preferred Reporting Items for Systematic Reviews and Meta-Analyses
LR+: Positive likelihood ratio
LR−: Negative likelihood ratio
ROC: Receiver operating characteristic
